# Cardiometabolic deaths in premenopausal black and white women

**DOI:** 10.1016/j.ajpc.2025.101050

**Published:** 2025-06-23

**Authors:** Rebecca Arden Harris, Sameed Ahmed M. Khatana, Judith A. Long

**Affiliations:** a Penn Cardiovascular Institute, University of Pennsylvania, Philadelphia, PA USA; b Leonard Davis Institute of Health Economics, University of Pennsylvania, Philadelphia, PA USA; c Department of Family Medicine and Community Health, Perelman School of Medicine, University of Pennsylvania, Philadelphia, PA USA; d Division of Cardiovascular Medicine, University of Pennsylvania, Philadelphia, PA USA; e Corporal Michael J. Crescenz VA Medical Center, Philadelphia, Pennsylvania, USA; f Division of General Internal Medicine, Perelman School of Medicine, University of Pennsylvania, Philadelphia, USA

**Keywords:** Cardiometabolic mortality, Black women, Premenopausal, Racial disparities, Years of life lost, Life table analysis

## Abstract

**Background:**

Black women in the United States bear a disproportionate burden of cardiometabolic disease (CMD) beginning in adolescence, while CMD mortality is often thought to emerge at menopause. This study estimates and compares CMD mortality risks in Black and White women aged 25-44 years.

**Methods:**

Using National Vital Statistics System data and life table methods, we estimated CMD mortality risk in hypothetical cohorts of non-Hispanic Black and White women aged 25-44, with cohort sizes matched to the 2022 U.S. population. We estimated cumulative risk and quantified excess mortality, proportional mortality, and years of life lost (YLL).

**Results:**

The cumulative CMD mortality was 0.90 % (95 % CI: 0.86 % to 0.93 %) for Black women and 0.33 % (95 % CI: 0.32 % to 0.34 %) for White women (risk ratio: 2.74, 95 % CI: 2.64 to 2.85). Of the 2,716 expected CMD deaths in the Black cohort, 1,735 or 63.89 % (95 % CI: 62.53 % to 65.24 %) were excess deaths. CMD accounted for 20.13 % (95 % CI: 19.45 % to 20.80 %) of deaths in Black women (rising from 7.8 % at age 25 to 27.6 % at age 44) versus 10.73 % (95 % CI: 10.40 % to 11.06 %) in White women (rising from 6.0 % to 15.6 %). Average YLL was 4.42 months (95 % CI: 4.40 to 4.44) for Black women and 1.70 months (95 % CI: 1.69 to 1.71) for White women.

**Conclusions:**

CMD mortality was higher in younger Black than White women and emerged well before menopause. Reducing these disparities requires earlier screening, improved healthcare access, and structural reforms.

## Highlights

• Cardiometabolic disease (CMD) mortality risk in premenopausal women is often overlooked.
• Period life tables provide reliable estimates of age-specific CMD mortality risk.
• One in 111 Black women aged 25 years is expected to die from CMD by age 45.
• One in 306 White women aged 25 years is expected to die from CMD by age 45.
• Six in ten CMD deaths of younger Black women were excess deaths relative to White women.

**Figure:**
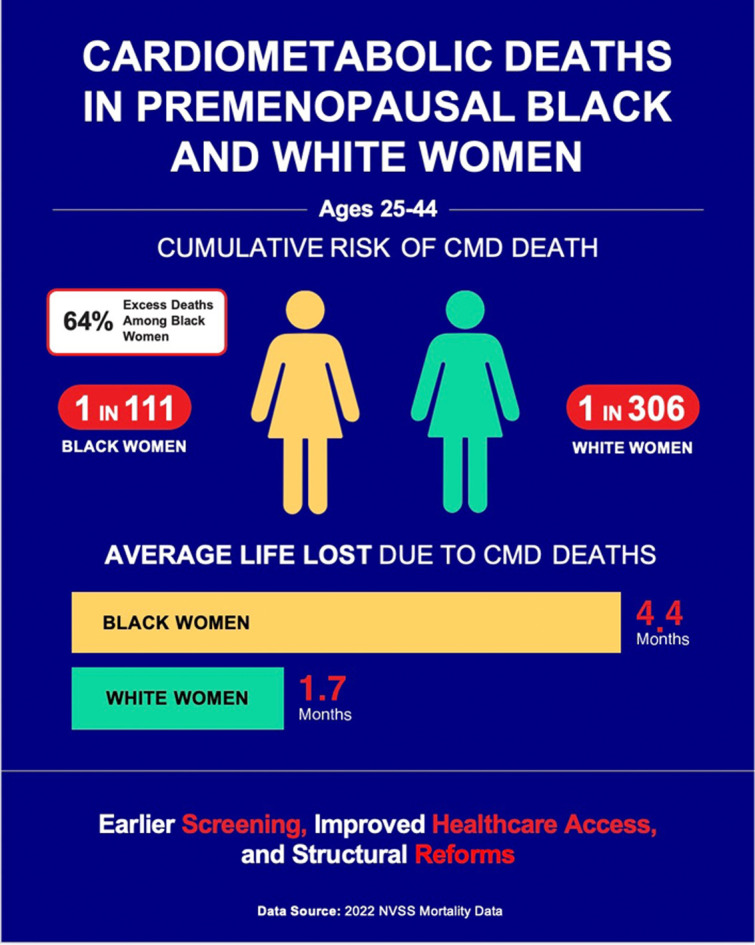
**Central illustration**: Risk of cardiometabolic death: Black vs. White premenopausal women.

## 1 Introduction

The disproportionate burden of cardiometabolic disease (CMD) among Black women in the United States, compared to White women, is well-documented [1]. CMD encompasses cardiovascular conditions such as hypertension, stroke, and coronary artery disease, as well as metabolic disorders like diabetes mellitus [2,3]. While recent research has highlighted the emergence of CMD among Black women during and after menopause [4], its clinical precursors are already pronounced in adolescence and young adulthood [5]. Young Black women typically have higher systolic blood pressure, elevated lipid and glucose levels, increased body mass index, and higher levels of inflammatory biomarkers compared to their White peers. Additionally, they often experience greater exposure to environmental stress and toxicants [6,7]. Black women also face unique health challenges, including higher rates of pregnancy-related complications, limited access to preventive care and nutritious food, and fewer opportunities for safe physical activity [8].

Despite the clear association between these risk factors and early onset of cardiometabolic morbidity [9], routine assessment of traditional cardiovascular risk factors is commonly recommended for adults aged 40 years and older [8,10]. This guidance particularly disadvantages premenopausal women, whose early cardiac symptoms often present differently than those typically seen in men and which informed traditional diagnostic criteria [11]. In populations with generally higher risk factors, such as young Black women, practice recommendations may overlook key opportunities for early screening and intervention or lack specific guidance for caring for at-risk groups [12]. Similarly, research into CMD mortality often overlooks younger age groups and women-specific risk factors [13], either by omitting younger people from the study sample or by calculating age-adjusted comparisons between Black and White populations, which effectively masks the impact on younger ages [14,15]. The focus on menopause as a key transition point in CMD risk—due to the loss of estrogen’s protective effects and the rise in metabolic and vascular changes—has further shifted attention toward midlife and older women, contributing to the neglect of premenopausal women despite their substantial burden of risk factors [16]. This oversight creates a notable gap in understanding the CMD mortality burden across the life cycle.

To address this gap, we used standard actuarial methods to estimate and compare premature CMD mortality among younger Black and White women in the United States. This study is the first to identify the precise ages at which cardiometabolic mortality disparities emerge between Black and White women, examining death risk by individual year of age from 25 to 44, rather than broad age categories (e.g., ‘under 55’) typically used in cardiovascular research [17]. We focus on premenopausal women because cardiometabolic disease in this group is often underdiagnosed and undertreated compared to men [11], reflecting distinct biological pathways, different disease manifestation, systemic gaps in preventive care, and historical underrepresentation in clinical research. Using cross-sectional national death certificate data, we constructed synthetic cohorts of Black and White women, and projected these cohorts forward from early adulthood to middle age, which allowed us to reliably estimate and compare CMD mortality. Addressing premature cardiovascular mortality in women is key to equitably improving life expectancy across the United States [18].

## 2 Methods

### 2.1 Study population

We used conventional period life table methods to estimate the risk of CMD death for non-Hispanic Black and White women aged 25-44 years [19], in line with our prior work [20]. A period life table estimates mortality outcomes for a hypothetical cohort based on the current age-specific probabilities of cardiometabolic death. In our hypothetical cohort, we followed 302,707 individuals, representing all 25-year-old Black women in the U.S., for 20 years beginning January 2022. We used the same procedures to estimate the risk of death for a comparison cohort of 1,121,345 White women, the population of 25-year-old White women in January 2022.

### 2.2 Life table construction

To build the tables, we calculated the number of individuals surviving from one year to the next by multiplying the cohort population size at each age, starting at age 25, by 1 minus the probability of death from all causes at that age. Age-specific all-cause death rates for non-Hispanic Black and White women were obtained from the National Vital Statistics System (NVSS) for 2022, the most recent year available [21]. We then applied the NVSS 2022 age-specific cardiometabolic death rates for Black and White women to the cohort populations to calculate the expected number of deaths, stratified by two groups of underlying causes: cardiovascular diseases (International Classification of Diseases, 10th Revision [ICD-10] codes I00-I69, encompassing heart disease, essential hypertension, hypertensive renal disease, and stroke) and metabolic diseases (ICD-10 codes E10-E14, representing diabetes) [22]. U.S. Census population estimates were adjusted from mid-year to January 1 to meet the life table requirement of cohort population counts at the start of each year.

### 2.3 Statistical analysis

We summed the expected number of cardiometabolic deaths from age 25 to 44 years and calculated the cumulative risk estimates, excess mortality (the difference between the Black and White estimates), proportional mortality (the percentage of all deaths attributed to a specific cause), and years of life lost (life expectancy minus age at death). Period life tables generate unbiased estimates because they are not influenced by the age distribution of the source populations.

Following standard practice in vital statistics and mortality research, the confidence intervals in our period life table analysis reflect stochastic (random) variation in observed death counts rather than sampling error, since we analyzed the complete population rather than a sample [23,24]. Standard errors and confidence intervals were estimated using the Wald method, which relies on approximate normality of estimators and accounts for random fluctuations in mortality over time and across populations [25]. This approach is well-suited to our study population of women aged 25-44, as it excludes the youngest and oldest ages where sparse data and skewed distributions can undermine normal approximations, and involves sufficiently frequent events to support reliable inference. For risk and rate ratios, percent excess deaths, and proportional mortality ratios, which are nonlinear functions computed from multiple estimates, we used the delta method to estimate confidence intervals, as it provides an approximation of the variance for nonlinear transformations of estimated rates [19,25].

To assess robustness, we re-estimated these parameters using pre-pandemic NVSS data from 2019 and compared the results to those from 2022. To further place this analysis into historical context, we graphed the trends in cardiometabolic death rates from 1999 to 2022 for Black and White women aged 25-44 years, based on the specified ICD-10 codes.

This study used anonymized publicly available data and was deemed exempt from IRB review under University of Pennsylvania guidelines.

## 3 Results

### 3.1 Cumulative risk

Of the 302,707 Black women aged 25 years in the initial cohort, the cumulative risk of cardiometabolic death before age 45 was 1 in 111 individuals or 0.90 % (95 % CI: 0.86 % to 0.93 %) (Table 1). The cumulative risk of death from metabolic diseases was 1 in 633 individuals or 0.16 % (95 % CI: 0.14 % to 0.17 %), and the cumulative risk of cardiovascular death was 1 in 135 individuals or 0.74 % (95 % CI: 0.71 % to 0.77 %).

**Table 1:** Life table for Black women ages 25-44: Cumulative risk of death and proportional mortality from cardiometabolic diseases*.

Age interval (Years)	2022 Black women all-cause mortality (Deaths/Population)	Hypothetical cohort of Black women in U.S. population alive at beginning of each age interval (N)	Expected deaths from cardiometabolic diseases (N)	Black Women Proportional Mortality: % of all deaths due to cardiometabolic diseases (%)	Expected deaths from metabolic diseases (N)	Black Women Proportional Mortality: % of all deaths due to metabolic diseases (%)	Expected deaths from cardiovascular diseases (N)	Black women Proportional Mortality: % of all deaths due to cardiovascular diseases (%)
25–26	0.001164	302,707	29	7.7	9	2.4	20	5.3
26–27	0.001248	302,355	40	10.8	10	2.7	30	8.1
27–28	0.001335	301,977	36	8.6	13	3.0	23	5.6
28–29	0.001419	301,574	46	10.2	15	3.3	31	7.0
29–30	0.001499	301,146	58	11.6	15	3.0	43	8.6
30–31	0.001578	300,695	64	11.6	14	2.5	50	9.1
31–32	0.001659	300,220	70	12.6	18	3.2	52	9.3
32–33	0.001748	299,722	75	13.0	13	2.2	63	10.8
33–34	0.001852	299,198	96	14.8	14	2.2	82	12.6
34–35	0.001978	298,644	121	18.6	23	3.5	99	15.1
35–36	0.002120	298,054	115	17.2	18	2.6	98	14.6
36-37	0.002277	297,422	131	18.4	24	3.3	107	15.1
37-38	0.002449	296,744	143	20.2	22	3.1	121	17.1
38-39	0.002628	296,018	187	25.6	37	5.1	150	20.6
39-40	0.002804	295,240	198	24.7	43	5.3	155	19.4
40-41	0.002992	294,412	210	24.3	31	3.6	179	20.7
41-42	0.003186	293,531	244	26.3	35	3.7	209	22.6
42-43	0.003366	292,596	282	28.9	51	5.2	231	23.7
43-44	0.003532	291,611	296	29.1	41	4.1	255	25.0
44-45	0.003698	290,581	274	27.6	34	3.4	240	24.1
	**Total:**		2,716		479		2,237	
**Cumulative risk (95% CI):**			0.90% (0.86 to 0.93)		0.16% (0.14 to 0.17)		0.74% (0.71 to 0.77)	
**100/cumulative risk:**			111		633		135	

⁎ Rounding.

For White women, the risks were markedly lower. Of the 1,121,345 White women aged 25 years in the initial cohort, the cumulative risk of cardiometabolic death before age 45 was 1 in 306 individuals or 0.33 % (95 % CI: 0.32 % to 0.34 %) (Table 2). The cumulative risk of death from metabolic diseases was 1 in 1,717 individuals or 0.06 % (95 % CI: 0.05 % to 0.06 %), and the cumulative risk of cardiovascular death was 1 in 372 individuals or 0.27 % (95 % CI: 0.26 % to 0.28 %).

**Table 2:** Life table for White women ages 25-44: Cumulative risk of death and proportional mortality from cardiometabolic diseases*.

Age interval (Years)	2022 White women all-cause mortality (Deaths/Population)	Hypothetical cohort of White women in U.S. population alive at beginning of each age interval (N)	Expected deaths from cardiometabolic diseases (N)	Proportional Mortality: % of all deaths due to cardiometabolic diseases (%)	Expected deaths from metabolic diseases (N)	Proportional Mortality: % of all deaths due to metabolic diseases (%)	Expected deaths from cardiovascular diseases (N)	Proportional Mortality: % of all deaths due to cardiovascular diseases (%)
25–26	0.000663	1,121,345	45	6.0	8.0	1.1	37.1	5.0
26–27	0.000732	1,120,602	55	6.9	14.0	1.8	41.0	5.2
27–28	0.000805	1,119,781	67	7.2	15.7	1.7	51.1	5.5
28–29	0.000879	1,118,880	81	8.0	12.6	1.2	68.6	6.8
29–30	0.000 0	1,117,896	71	6.2	20.8	1.8	50.2	4.4
30–31	0.001020	1,116,834	70	5.8	15.6	1.3	54.2	4.5
31–32	0.001091	1,115,695	99	7.2	18.7	1.4	80.2	5.9
32–33	0.001164	1,114,478	121	8.1	29.9	2.0	91.4	6.1
33–34	0.001239	1,113,181	112	7.1	28.8	1.8	83.6	5.3
34–35	0.001321	1,111,801	145	8.9	37.2	2.3	107.8	6.6
35–36	0.001408	1,110,333	157	9.5	21.9	1.3	135.2	8.1
36-37	0.001499	1,108,769	185	10.0	35.3	1.9	150.2	8.1
37-38	0.001592	1,107,107	206	10.7	38.7	2.0	167.4	8.7
38-39	0.001682	1,105,345	226	10.8	45.9	2.2	180.1	8.6
39-40	0.001772	1,103,486	249	11.2	45.1	2.0	203.7	9.2
40-41	0.001874	1,101,530	286	12.2	52.2	2.2	233.3	10.0
41-42	0.001985	1,099,466	323	13.2	48.1	2.0	275.0	11.2
42-43	0.002100	1,097,284	368	14.5	52.1	2.1	316.1	12.5
43-44	0.002218	1,094,979	387	15.0	48.1	1.9	338.9	13.1
44-45	0.002350	1,092,551	413	15.6	64.5	2.4	348.3	13.2
	**Tota**l:		3,667		653		3,013	
**Cumulative risk (95** % **CI):**			0.33 % (0.32 to 0.34)		0.06 % (0.05 to 0.06)		0.27 % (0.26 to 0.28)	
**100/cumulative risk:**			306		1,717		372	

⁎ Rounding.

The Black-to-White risk ratio for cardiometabolic deaths was 2.74 (95 % CI: 2.64 to 2.85). For metabolic deaths, the risk ratio was 2.71 (95 % CI: 1.29 to 5.68). For cardiovascular deaths, the risk ratio was 2.75 (95 % CI: 2.65 to 2.86).

### 3.2 Excess deaths

Of the 2,716 expected deaths among Black women aged 25-44, 1,735 or 63.89 % (95 % CI: 62.53 % to 65.24 %) were excess deaths, relative to what would be expected if Black women had the same age-specific mortality rates as White women. Excess metabolic deaths were estimated at 304 of the expected 479 metabolic deaths, or 63.44 % (95 % CI: 59.22 % to 67.66 %), and excess cardiovascular deaths were estimated at 1,431 of the expected 2,237 deaths, or 63.94 % (95 % CI: 62.01 % to 65.88 %).

### 3.3 Proportional mortality

This measure calculates the contribution of CMD to total mortality by dividing the number of CMD deaths by the total number of deaths from all causes, then multiplying by 100 [26]. Among Black women, cardiometabolic diseases accounted for 20.13 % of all deaths (95 % CI: 19.45 % to 20.80 %), increasing from 7.75 % at age 25 to 27.59 % at age 44. Among White women, cardiometabolic diseases accounted for 10.73 % of all deaths (95 % CI: 10.40 % to 11.06 %), increasing from 6.03 % at age 25 to 15.63 % at age 44. The Black-to-White proportional mortality ratio was 1.88 (95 % CI: 1.79 to 1.96).

For metabolic diseases, Black women had a proportional mortality of 3.55 % (95 % CI: 3.24 % to 3.86 %), increasing from 2.40 % to 3.45 %. White women had a proportional mortality of 1.91 % (95 % CI: 1.77 % to 2.06 %), increasing from 1.07 % to 2.44 %. The Black-to-White proportional mortality ratio was 1.86 (95 % CI: 1.65 to 2.09).

For cardiovascular diseases, Black women had a proportional mortality of 16.58 % (95 % CI: 15.95 % to 17.20 %), increasing from 5.34 % to 24.14 %. White women had a proportional mortality of 8.81 % (95 % CI: 8.51 % to 9.12 %), increasing from 4.96 % to 13.18 %. The Black-to-White proportional mortality ratio was 1.88 (95 % CI: 1.79 to 1.98).

**Fig. 1:**
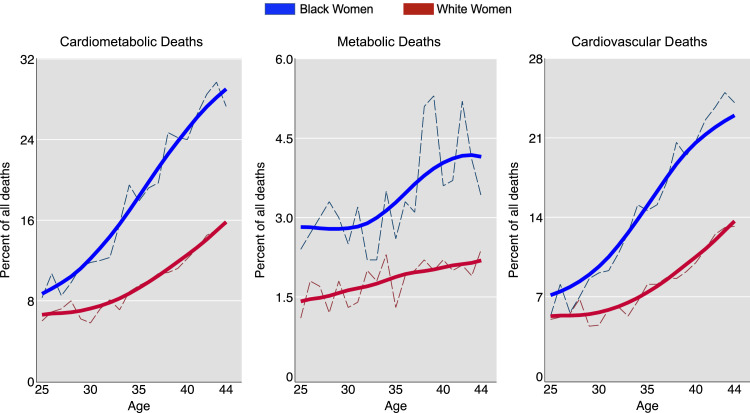
Proportional mortality (percent of all deaths due to cardiometabolic diseases) for Black and White women ages 25-44 years*.

Compared to cardiovascular proportional mortality, metabolic proportional mortality increased less with age (Fig. 1).

### 3.4 Years of life lost (YLL)

We estimated YLL, calculated as life expectancy minus age at death from CMD, for each age interval over the 25-44-year age range (Table 3). Black women experienced a total of 111,458 YLL (95 % CI: 110,894 to 112,021), with an average loss of 4.42 months (95 % CI: 4.40 to 4.44) of life per person in the cohort. White women had a total of 158,905 YLL (95 % CI: 158,169 to 159,641), averaging 1.70 months (95 % CI: 1.69 to 1.71) of lost life per person. Black women lost 2.60 times as many months of life per cohort member compared to White women (95 % CI: 2.58 to 2.62).

**Table 3:** Years of life lost (YLL) for Black and White women ages 25-44 from cardiometabolic death*.

Age interval (Years)	Hypothetical cohort of Black women in U.S. population alive at beginning of each age interval (N)	Expected deaths from CMD among Black women (N)	Expected years of additional life among Black women (Years)	YLL due to CMD among Black women (Years)	Hypothetical cohort of White women in U.S. population alive at beginning of each age interval (N)	Expected deaths from CMD among White women (N)	Expected years of additional life among White women (Years)	YLL due to CMD among White women (Years)
25–26	302,707	29	53.0	1,548	1,121,345	45	55.8	2,519
26–27	302,355	40	52.1	2,098	1,120,602	55	54.9	3,017
27–28	301,977	36	51.1	1,822	1,119,781	67	53.9	3,603
28–29	301,574	46	50.2	2,304	1,118,880	81	52.9	4,292
29–30	301,146	58	49.3	2,853	1,117,896	71	52.0	3,696
30–31	300,695	64	48.4	3,110	1,116,834	70	51.0	3,559
31–32	300,220	70	47.4	3,321	1,115,695	99	50.1	4,956
32–33	299,722	75	46.5	3,507	1,114,478	121	49.1	5,957
33–34	299,198	96	45.6	4,381	1,113,181	112	48.2	5,417
34–35	298,644	121	44.7	5,425	1,111,801	145	47.3	6,859
35–36	298,054	115	43.8	5,055	1,110,333	157	46.3	7,273
36-37	297,422	131	42.8	5,608	1,108,769	185	45.4	8,420
37-38	296,744	143	41.9	5,995	1,107,107	206	44.5	9,174
38-39	296,018	187	41.0	7,665	1,105,345	226	43.5	9,831
39-40	295,240	198	40.2	7,942	1,103,486	249	42.6	10,599
40-41	294,412	210	39.3	8,247	1,101,530	286	41.7	11,907
41-42	293,531	244	38.4	9,369	1,099,466	323	40.7	13,150
42-43	292,596	282	37.5	10,560	1,097,284	368	39.8	14,651
43-44	291,611	296	36.6	10,832	1,094,979	387	38.0	14,709
44-45	290,581	274	35.8	9,816	1,092,551	413	37.1	15,316
	**Total YLL:**			111,458			158,905	
**Average YLL:** **(95** % **CI):**			4.42 months (4.40 to 4.44)				1.70 months (1.69 to 1.71)	

⁎ Rounding.

### 3.5 Sensitivity analysis

The 2019 pre-pandemic results were comparable to the 2022 post-pandemic results (see Supplemental Tables S1, S2, and S3, and Table 4).

**Table 4:** Comparison of cardiometabolic mortality risks among Black and White women ages 25-44, 2019 and 2022*.

Measure	2019	2022
	*Estimate (95* % *CI)*	*Estimate (95* % *CI)*
**Cumulative Risk of Death (Before Age 45)**		
*Black Women*		
Cardiometabolic Deaths	0.76 % (0.73 to 0.79)	0.90 % (0.86 to 0.93)
Metabolic Deaths	0.12 % (0.11 to 0.13)	0.16 % (0.14 to 0.17)
Cardiovascular Deaths	0.64 % (0.61 to 0.67)	0.74 % (0.71 to 0.77)
*White Women*		
Cardiometabolic Deaths	0.29 % (0.28 to 0.30)	0.33 % (0.32 to 0.34)
Metabolic Deaths	0.05 % (0.04 to 0.05)	0.06 % (0.05 to 0.06)
Cardiovascular Deaths	0.24 % (0.24 to 0.25)	0.27 % (0.26 to 0.28)
*Black-to-White Cumulative Risk Ratios*		
Cardiometabolic Deaths	2.63 (2.49 to 2.77)	2.74 (2.64 to 2.85)
Metabolic Deaths	2.61 (2.28 to 2.98)	2.71 (1.29 to 5.68)
Cardiovascular Deaths	2.63 (2.48 to 2.79)	2.75 (2.65 to 2.86)
**Excess Deaths (Percent) in Black Women Ages 25–44**		
Excess Cardiometabilic Deaths	62.11 % (60.09 to 64.13)	63.89 % (62.53 to 65.24)
Excess Metabolic Deaths	61.87 % (56.80 to 66.94)	63.44 % (59.22 to 67.66)
Excess Cardiovascular Deaths	62.16 % (59.96 to 64.36)	63.94 % (62.01 to 65.88)
**Proportional Mortality (% CMD of All-Cause Deaths)**		
*Black Women*		
Cardiometabolic	24.95 % (24.10 to 25.80)	20.13 % (19.45 to 20.80)
Metabolic	3.99 % (3.60 to 4.37)	3.55 % (3.24 to 3.86)
Cardiovascular	20.96 % (20.16 to 21.76)	16.58 % (15.95 to 17.20)
*White Women*		
Cardiometabolic	11.64 % (11.26 to 12.01)	10.73 % (10.40 to 11.06)
Metabolic	1.87 % (1.71 to 2.03)	1.91 % (1.77 to 2.06)
Cardiovascular	9.77 % (9.42 to 10.11)	8.81 % (8.51 to 9.12)
*Black-to-White Proportional Mortality Ratios*		
Cardiometabolic	2.14 (2.05 to 2.25)	1.88 (1.79 to 1.96)
Metabolic	2.13 (1.88 to 2.43)	1.86 (1.65 to 2.09)
Cardiovascular	2.15 (2.04 to 2.26)	1.88 (1.79 to 1.98)
**Years of Lost Life (YLL): Cardiometabolic Deaths Ages 25-44**		
Black Women (Years)	104,581 (104,000 to 105,162)	111,458 (110,894 to 112,021)
Average Across Black Women Cohort (Months)	3.88 (3.86 to 3.90)	4.42 (4.40 to 4.44)
White Women (Years)	147,502 (146,812 to 148,192)	158,905 (158,169 to 159,641)
Average Across White Women Cohort (Months)	1.56 (1.55 to 1.56)	1.70 (1.69 to 1.71)
Black to White Ratio of Cohort Averages	2.49 (2.47 to 2.51)	2.60 (2.58 to 2.62)

⁎ Rounding.

## 4 Discussion

This study reveals striking differences in the cumulative risk of cardiometabolic mortality between Black and White women. Between age 25 and 44, Black women are 2.7 times more likely to die from CMD before middle age, with risks of 1 in 111 for Black women versus 1 in 306 for White women. Notably, CMD becomes a leading contributor to mortality earlier in adulthood for Black women than for White women. Both groups show low percentages of CMD deaths at age 25, but by age 45, 27 % of deaths in Black women are from CMD compared to 16 % in White women. CMD also shortened the average lifespan of younger Black women by 4.4 months, compared to 1.7 months for White women. These findings were similar in our robustness check using 2019 pre-pandemic data.

Our life table analysis offers several contributions to understanding racial health disparities. First, by focusing specifically on premenopausal women aged 25-44, we characterize mortality patterns in a group that has been understudied despite facing increasing cardiometabolic risk. Second, our age-specific approach establishes that racial disparities in cardiometabolic mortality emerge early and intensify with age, a finding that has important implications for the timing of preventive interventions. Third, we quantify the magnitude of disparity (2.7-fold higher mortality among Black women by age 44) and translate this into clinically meaningful metrics such as cumulative risk or 1-in-X risk of death, mortality ratio, excess mortality, proportional mortality, and years of life lost. These estimates may provide useful targets for health equity initiatives and baseline parameters for evaluating policy effectiveness.

In this paper, we conceptualize race as a social construct that serves as a proxy for the impact of systemic racism [27]. The mortality disparities we observed reflect the cumulative effects of social, historical, economic, and environmental factors [28]. The interpretation of race indicators in medical contexts is a sensitive issue. For example, the American Heart Association (AHA) excluded race from its new CVD risk calculator to avoid perpetuating the misconception that race is a biological or genetic determinant [29]. Instead, the AHA calculator provides users with an option: a zip code-based “Social Deprivation Index” (SDI), which is primarily driven by the percentage of residents living in poverty [30,31]. While the AHA-SDI focus on poverty is understandable, its disregard of race as a social signifier in the United States is a concern. It overlooks the biases that race elicits, distinct from poverty but pervasive in the daily interactions and life opportunities of Black women, with potential severe impacts on cardiometabolic health over time [32]. However, integrating race or racism into risk assessment tools faces practical challenges: area-level measures risk ecological fallacies (assuming group-level data applies to individuals), while individual-level assessments in clinical settings raise ethical concerns about stigmatization. Though the biological mechanisms linking racial adversity and chronic stress to cardiometabolic risks are complex and not fully understood, emerging research identifies potential pathways such as pro-inflammatory signaling, cellular dysfunction, telomere shortening, and epigenetic aging [[33], [34], [35]].

Our findings have clear implications for clinical care and public health policy. First, risk-based screening guidelines should be recalibrated to reflect the earlier age of CMD onset in Black women. Screening for hypertension, diabetes, and lipid disorders, as well as behavioral risk factors like smoking, should begin before age 30 in this population. Second, health systems should implement targeted, culturally responsive prevention efforts, including community-based screening initiatives, patient navigation programs, and clinical decision support tools that incorporate social risk factors [36]. For example, church-based health programs and peer-led interventions have been effective in increasing preventive care uptake among Black women [37]. Third, policy responses should address the root causes of early CMD mortality by expanding access to Medicaid, investing in Federally Qualified Health Centers (FQHCs), and directing funds to improve the built environment and social infrastructure in historically disinvested Black communities [38]. Urban planning policies that support walkability, affordable healthy food access, and safe recreational spaces can also reduce cardiometabolic risk [8,39].

Reducing CMD disparities will require sustained investment in both upstream structural reform and downstream clinical action. Health systems, payers, and policymakers must work collaboratively to prioritize young Black women for early screening, prevention, and care. Future research should evaluate the impact of integrating social and racial adversity metrics into cardiovascular risk prediction and test interventions that address these determinants across the life course [29,40].

### 4.1 Strengths and limitations

Period life tables offer a standardized approach to measuring age-specific mortality under current conditions [19,25,41]. By simulating hypothetical cohorts exposed to prevailing mortality rates, they enable direct estimation and comparison of mortality burdens. This makes them particularly useful for assessing disparities across demographic groups using complete, population-level data [41]. Methodologically, they provide internally consistent mortality metrics, support comparisons across populations with varying age structures, and offer flexibility in defining the age range for analysis [25]. Additionally, their outputs—such as 1-in-X risk, excess deaths, and average months of life lost—are easily interpretable by clinical, public health, and community and policy stakeholders.

However, period life tables are not designed for individual risk prediction and do not incorporate clinical or behavioral covariates required for causal inference, such as blood pressure, lipid levels, HbA1c, medication use, smoking history, or kidney function. This reflects an inherent difference in purpose; life tables are a powerful tool for estimating population-level mortality and differential burden using national data. They provide a clear picture of current mortality patterns by showing the cumulative effects of age-specific death rates from a given year [24], but they cannot account for individual-level risk factors or predict how mortality rates might evolve over time, which can affect the accuracy of long-term projections.

In the broader context of epidemiological research, individual-level approaches aim to identify causal relationships and evaluate interventions, often using clinical or behavioral data. Population-level approaches examine how disease and mortality distribute across groups and over time, identifying patterns, informing policy, and monitoring disparities [42]. These complementary approaches strengthen scientific understanding: individual models test mechanistic hypotheses and treatment effects, while population-level tools specify the scale, scope, and structure of disparities to inform public health policies and interventions [43].

Lastly, we acknowledge the potential misclassification of cause of death in death certificate data, particularly in the context of comorbidities or diagnostic uncertainty. To mitigate this limitation, we used broad ICD-10 code groupings consistent with prior cardiometabolic research [44].

## 5 Conclusions

**Fig. 2:**
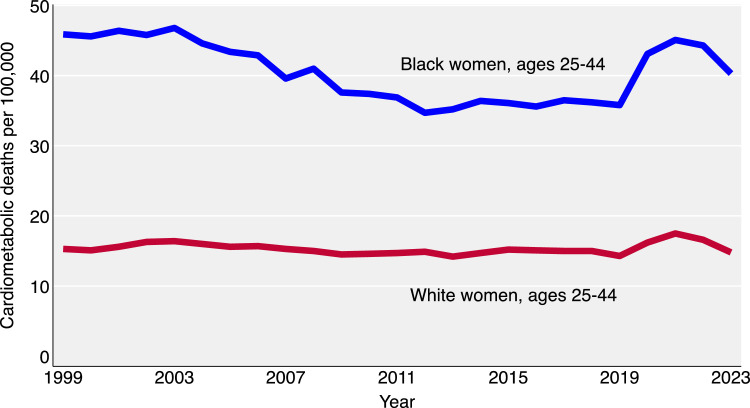
Black and White female cardiometabolic mortality rate, 1999-2023.

Since 2012, improvements in CMD mortality among Black women in the U.S. have stalled (Fig. 2). The disruptions of the COVID-19 pandemic in 2020–2021 caused temporary spikes in mortality, especially for young Black women, but rates are now beginning to return to pre-pandemic levels. Our life table analysis reveals how CMD mortality rates that are initially comparable between Black and White women at age 25 diverge progressively with age, with Black women being 2.7 times more likely to die from CMD before middle age. These findings underscore the urgent need for earlier, tailored screening, targeted prevention, and structural policy reforms to reduce CMD mortality in this high-risk group. Intervening early, before the onset of symptomatic disease, represents both a clinical imperative and a public health opportunity.

## Funding

Dr. Harris' work is supported by a grant from the 10.13039/100000026National Institute on Drug Abuse (K23 DA054157). Dr. Khatana's work is supported by grants from the 10.13039/100000050National Heart, Lung, and Blood Institute (K23 HL153772, R01 HL171157).

## CRediT authorship contribution statement

**Rebecca Arden Harris:** Writing – review & editing, Writing – original draft, Methodology, Formal analysis, Data curation, Conceptualization. **Sameed Ahmed M. Khatana:** Writing – review & editing, Writing – original draft, Methodology. **Judith A. Long:** Writing – review & editing, Writing – original draft, Methodology.

## Declaration of competing interest

The authors declare that they have no known competing financial interests or personal relationships that could have appeared to influence the work reported in this paper.

## Appendix Supplementary materials

Image, application 1
