# Supplementary material for: Cardiometabolic deaths in premenopausal black and white women
Source: Am J Prev Cardiol. 2025 Jun 23;23:101050. doi: 10.1016/j.ajpc.2025.101050 (PMC12269981; doi:10.1016/j.ajpc.2025.101050)
Supplement: Supplementary file 1 [file mmc1.docx]

**Cardiometabolic Deaths in Premenopausal Black and White Women**

**Supplement**

**Sensitivity Analysis using 2019 data:**

**Table S1.** Life table for Black women ages 25-44: Cumulative risk of death and proportional mortality from cardiometabolic diseases

**Table S2.** Life table for White women ages 25-44: Cumulative risk of death and proportional mortality from cardiometabolic diseases

**Table S3.** Years of life lost (YLL) for Black and White women ages 25-44 from cardiometabolic death

**Table S1.** Life table for Black women ages 25-44: Cumulative risk of death and proportional mortality from cardiometabolic diseases*

| Age interval  (Years) | 2019  Black women  all-cause mortality  (Deaths/Population) | Hypothetical cohort of Black women in U.S. population alive at beginning of each age interval  (N) | Black women: Expected  deaths from cardiometabolic diseases  (N) | Black women Proportional  Mortality:  % of all  deaths due to cardiometabolic diseases  (%) | Expected  deaths from metabolic diseases  (N) | Black women Proportional  Mortality:  % of all deaths due to metabolic diseases  (%) | Expected  deaths from cardiovascular diseases  (N) | Black women Proportional  Mortality:  % of all deaths due to cardiovascular diseases  (%) |
| --- | --- | --- | --- | --- | --- | --- | --- | --- |
| 25–26 | 0.000831 | 323,605 | 30 | 11.3 | 6 | 2.19 | 24 | 9.11 |
| 26–27 | 0.000844 | 323,336 | 34 | 10.6 | 5 | 1.47 | 29 | 9.14 |
| 27–28 | 0.000876 | 323,063 | 41 | 12.7 | 14 | 4.34 | 27 | 8.40 |
| 28–29 | 0.000938 | 322,780 | 34 | 10.6 | 5 | 1.67 | 29 | 8.91 |
| 29–30 | 0.001028 | 322,477 | 43 | 13.0 | 7 | 2.21 | 36 | 10.78 |
| 30–31 | 0.001134 | 322,145 | 70 | 18.2 | 15 | 4.05 | 54 | 14.17 |
| 31–32 | 0.001245 | 321,780 | 70 | 18.1 | 14 | 3.73 | 56 | 14.38 |
| 32–33 | 0.001360 | 321,379 | 91 | 22.3 | 13 | 3.15 | 78 | 19.16 |
| 33–34 | 0.001469 | 320,942 | 93 | 20.6 | 15 | 3.35 | 78 | 17.24 |
| 34–35 | 0.001572 | 320,471 | 109 | 23.8 | 22 | 4.72 | 87 | 19.10 |
| 35–36 | 0.001682 | 319,967 | 110 | 23.3 | 17 | 3.57 | 93 | 19.73 |
| 36-37 | 0.001803 | 319,429 | 93 | 18.3 | 18 | 3.48 | 75 | 14.81 |
| 37-38 | 0.001921 | 318,853 | 127 | 23.7 | 23 | 4.30 | 104 | 19.44 |
| 38-39 | 0.002037 | 318,241 | 171 | 28.4 | 30 | 4.95 | 141 | 23.48 |
| 39-40 | 0.002156 | 317,592 | 197 | 30.0 | 28 | 4.22 | 169 | 25.80 |
| 40-41 | 0.002289 | 316,908 | 200 | 31.4 | 23 | 3.55 | 178 | 27.84 |
| 41-42 | 0.002440 | 316,182 | 225 | 34.5 | 41 | 6.29 | 184 | 28.22 |
| 42-43 | 0.002606 | 315,411 | 211 | 30.5 | 24 | 3.41 | 187 | 27.07 |
| 43-44 | 0.002781 | 314,589 | 240 | 32.0 | 34 | 4.57 | 206 | 27.44 |
| 44-45 | 0.002967 | 313,714 | 284 | 37.4 | 42 | 5.48 | 242 | 31.93 |
|  | **Total:** | | 2,472 |  | 395 |  | 2,077 |  |
| **Cumulative risk (95% CI):** | | | 0.76% (0.73 to 0.79) | | 0.12% (0.11 to 0.13) | | 0.64% (0.61 to 0.67) | |
| **100/cumulative risk:** | | | 131 | | 833 | | 156 | |

*Rounding

**Table S2.**

Life table for White women ages 25-44: Cumulative risk of death and proportional mortality from cardiometabolic diseases*

| Age interval  (Years) | 2019  White women  all-cause mortality  (Deaths/Population) | Hypothetical cohort of White women in U.S. population alive at beginning of each age interval  (N) | Expected  deaths from cardiometabolic diseases  (N) | White women Proportional  Mortality:  % of all  deaths due to cardiometabolic diseases  (%) | Expected  deaths from metabolic­­­ diseases  (N) | White women Proportional  Mortality:  % of all deaths due to metabolic diseases  (%) | Expected  deaths from cardiovascular diseases  (N) | White women Proportional  Mortality:  % of all deaths due to cardiovascular diseases  (%) |
| --- | --- | --- | --- | --- | --- | --- | --- | --- |
| 25–26 | 0.000613 | 1,137,104 | 38 | 5.3 | 8 | 1.1 | 30 | 4.2 |
| 26–27 | 0.000658 | 1,136,407 | 51 | 6.5 | 10 | 1.3 | 41 | 5.2 |
| 27–28 | 0.000706 | 1,135,659 | 66 | 7.5 | 8 | 0.9 | 58 | 6.5 |
| 28–29 | 0.000761 | 1,134,857 | 52 | 5.3 | 6 | 0.6 | 46 | 4.7 |
| 29–30 | 0.000822 | 1,133,994 | 85 | 8.1 | 15 | 1.4 | 70 | 6.7 |
| 30–31 | 0.000887 | 1,133,062 | 88 | 7.7 | 15 | 1.4 | 73 | 6.4 |
| 31–32 | 0.000952 | 1,132,057 | 91 | 7.8 | 8 | 0.7 | 83 | 7.1 |
| 32–33 | 0.001017 | 1,130,979 | 111 | 9.1 | 20 | 1.7 | 90 | 7.4 |
| 33–34 | 0.001080 | 1,129,829 | 119 | 8.5 | 20 | 1.4 | 99 | 7.0 |
| 34–35 | 0.001141 | 1,128,608 | 115 | 8.5 | 21 | 1.5 | 95 | 7.0 |
| 35–36 | 0.001208 | 1,127,321 | 130 | 9.1 | 20 | 1.4 | 111 | 7.7 |
| 36-37 | 0.001279 | 1,125,959 | 156 | 10.5 | 29 | 1.9 | 127 | 8.6 |
| 37-38 | 0.001341 | 1,124,519 | 167 | 10.4 | 23 | 1.4 | 144 | 9.0 |
| 38-39 | 0.001392 | 1,123,011 | 207 | 11.7 | 26 | 1.5 | 181 | 10.2 |
| 39-40 | 0.001440 | 1,121,448 | 246 | 13.2 | 52 | 2.8 | 193 | 10.4 |
| 40-41 | 0.001497 | 1,119,833 | 258 | 14.3 | 40 | 2.2 | 218 | 12.1 |
| 41-42 | 0.001574 | 1,118,156 | 272 | 14.9 | 45 | 2.5 | 227 | 12.4 |
| 42-43 | 0.001675 | 1,116,396 | 302 | 16.2 | 46 | 2.4 | 257 | 13.7 |
| 43-44 | 0.001801 | 1,114,526 | 330 | 16.8 | 49 | 2.5 | 281 | 14.2 |
| 44-45 | 0.001947 | 1,112,519 | 423 | 19.8 | 68 | 3.2 | 354 | 16.6 |
|  | **Tota**l: | | 3,307 |  | 531 |  | 2,776 | |
| **Cumulative risk (95% CI):** | | | 0.29% (0.28 to 0.30) | | 0.05% (0.04 to 0.5) | | 0.24% (0.24 to 0.25) | |
| **100/cumulative risk:** | | | 345 | | 2,128 |  | 410 | |

*Rounding

**Table S3.**

Years of life lost (YLL) for Black and White women ages 25-44 from cardiometabolic death*

| Age interval  (Years) | Hypothetical cohort of Black women in U.S. population alive at beginning of each age interval  (N) | Expected  deaths  from CMD among Black women  (N) | Expected  years of additional life among Black women  (Years) | YLL  due to  CMD  among  Black women  (Years) | | Hypothetical cohort of White women in U.S. population alive at beginning of each age interval  (N) | Expected  deaths  from CMD among White women  (N) | Expected  years of additional life among White women  (Years) | YLL  due to  CMD among White women  (Years) |
| --- | --- | --- | --- | --- | --- | --- | --- | --- | --- |
| 25–26 | 323,605 | 30 | 54.5 | 1,650 | | 1,137,104 | 38 | 56.9 | 2,142 |
| 26–27 | 323,336 | 34 | 53.5 | 1,829 | | 1,136,407 | 51 | 56.0 | 2,873 |
| 27–28 | 323,063 | 41 | 52.5 | 2,134 | | 1,135,659 | 66 | 55.0 | 3,632 |
| 28–29 | 322,780 | 34 | 51.6 | 1,774 | | 1,134,857 | 52 | 54.0 | 2,818 |
| 29–30 | 322,477 | 43 | 50.6 | 2,169 | | 1,133,994 | 85 | 53.1 | 4,516 |
| 30–31 | 322,145 | 70 | 49.7 | 3,459 | | 1,133,062 | 88 | 52.1 | 4,582 |
| 31–32 | 321,780 | 70 | 48.7 | 3,404 | | 1,132,057 | 91 | 51.2 | 4,662 |
| 32–33 | 321,379 | 91 | 47.8 | 4,330 | | 1,130,979 | 111 | 50.2 | 5,565 |
| 33–34 | 320,942 | 93 | 46.9 | 4,354 | | 1,129,829 | 119 | 49.3 | 5,859 |
| 34–35 | 320,471 | 109 | 45.9 | 5,006 | | 1,128,608 | 115 | 48.3 | 5,576 |
| 35–36 | 319,967 | 110 | 45.0 | 4,938 | | 1,127,321 | 130 | 47.4 | 6,174 |
| 36-37 | 319,429 | 93 | 44.1 | 4,091 | | 1,125,959 | 156 | 46.4 | 7,217 |
| 37-38 | 318,853 | 127 | 43.2 | 5,507 | | 1,124,519 | 167 | 45.5 | 7,578 |
| 38-39 | 318,241 | 171 | 42.2 | 7,223 | | 1,123,011 | 207 | 44.6 | 9,249 |
| 39-40 | 317,592 | 197 | 41.3 | 8,120 | | 1,121,448 | 246 | 43.6 | 10,708 |
| 40-41 | 316,908 | 200 | 40.4 | 8,090 | | 1,119,833 | 258 | 42.7 | 11,002 |
| 41-42 | 316,182 | 225 | 39.5 | 8,874 | | 1,118,156 | 272 | 41.7 | 11,347 |
| 42-43 | 315,411 | 211 | 38.6 | 8,141 | | 1,116,396 | 302 | 40.8 | 12,342 |
| 43-44 | 314,589 | 240 | 37.7 | 9,053 | | 1,114,526 | 330 | 39.9 | 13,180 |
| 44-45 | 313,714 | 284 | 36.8 | 10,435 | | 1,112,519 | 423 | 39.0 | 16,480 |
|  | **Total YLL:** | |  | 104,582 |  | |  | 147,504 | |
| **Average YLL:** | | | 3.88 months | | | |  | 1.56 months | |

*Rounding
